# Role of *N-*Glycosylation in FcγRIIIa interaction with IgG

**DOI:** 10.3389/fimmu.2022.987151

**Published:** 2022-09-09

**Authors:** Julie Van Coillie, Morten A. Schulz, Arthur E. H. Bentlage, Noortje de Haan, Zilu Ye, Dionne M. Geerdes, Wim J. E. van Esch, Lise Hafkenscheid, Rebecca L. Miller, Yoshiki Narimatsu, Sergey Y. Vakhrushev, Zhang Yang, Gestur Vidarsson, Henrik Clausen

**Affiliations:** ^1^ Copenhagen Center for Glycomics, Department of Cellular and Molecular Medicine, Faculty of Health Sciences, University of Copenhagen, Copenhagen, Denmark; ^2^ Department of Experimental Immunohematology, Sanquin Research, Amsterdam, Netherlands; ^3^ Department of Biomolecular Mass Spectrometry and Proteomics, Utrecht Institute for Pharmaceutical Sciences and Bijvoet Center for Biomolecular Research, Utrecht University, Utrecht, Netherlands; ^4^ Sanquin Reagents, Amsterdam, Netherlands; ^5^ GlycoDisplay ApS, Copenhagen, Denmark

**Keywords:** Fc gamma receptors, CD16a, mAbs, IgG, glycoengineering, *N-*glycosylation, glycosyltransferases, surface plasmon resonance

## Abstract

Immunoglobulins G (IgG) and their Fc gamma receptors (FcγRs) play important roles in our immune system. The conserved *N-*glycan in the Fc region of IgG1 impacts interaction of IgG with FcγRs and the resulting effector functions, which has led to the design of antibody therapeutics with greatly improved antibody-dependent cell cytotoxicity (ADCC) activities. Studies have suggested that also *N-*glycosylation of the FcγRIII affects receptor interactions with IgG, but detailed studies of the interaction of IgG1 and FcγRIIIa with distinct *N*-glycans have been hindered by the natural heterogeneity in *N-*glycosylation. In this study, we employed comprehensive genetic engineering of the *N-*glycosylation capacities in mammalian cell lines to express IgG1 and FcγRIIIa with different *N-*glycan structures to more generally explore the role of *N-*glycosylation in IgG1:FcγRIIIa binding interactions. We included FcγRIIIa variants of both the 158F and 158V allotypes and investigated the key *N-*glycan features that affected binding affinity. Our study confirms that afucosylated IgG1 has the highest binding affinity to oligomannose FcγRIIIa, a glycan structure commonly found on Asn162 on FcγRIIIa expressed by NK cells but not monocytes or recombinantly expressed FcγRIIIa.

## Introduction

Immunoglobulin G (IgG) consists of both an antigen-binding variable Fab region and an Fc region which allows antibodies to activate the complement system through C1q binding or activate effector cells through Fc gamma Receptor (FcγR) or CD16a binding. This interaction is influenced by both the IgG subclass and the IgG-Fc *N-*glycan composition at the conserved Asn297 site. The IgG1-Fc Asn297 *N-*glycan site is partially masked from the *N-*glycan glycosyltransferases and together with the glycosyltransferase substrate specificity and glycan-peptide backbone interactions, this results in a complex-type, biantennary, core-fucosylated *N-*glycan with partially incomplete galactosylation and α2-6 sialylation (1–5). The IgG1-Fc core fucose interferes with binding to FcγRIII and IgG1 lacking this core fucose, hereafter called afucosylated IgG1, has an increased affinity up to 40 times to FcγRIII (6–8). Interestingly, afucosylated IgG1 is generally low abundant in circulating IgG, but high levels of antigen-specific afucosylated IgG1 have been observed in several conditions against membrane-embedded epitopes, such as alloimmune responses to blood cells, malaria and enveloped viruses including; HIV, Dengue, and SARS-CoV-2 (9–15). In addition to core fucosylation, IgG-Fc galactosylation further enhances the affinity to FcγRIII (8, 16, 17).

The FcγRIIIa receptor is an activating IgG receptor of medium-high affinity (K_D_≈10-400 nM), mainly expressed on NK cells, macrophages, and monocytes (18–20). It has been shown that the binding strength of IgG1 and IgG3 to FcγRIIIa directly correlates with antibody-dependent cellular cytotoxicity (ADCC) and therapeutic outcome (21–25). There are two common allotypes: FcγRIIIa-158V and -158F. FcγRIIIa-158V has a 5-fold increased affinity to IgG1 in comparison to the 158F allotype (8), the latter being the dominant allele in humans (26–28). This glycoprotein has five *N-*glycan sites (Asn38, Asn45, Asn74, Asn162, and Asn169) of which mainly Asn162 influences IgG1 binding (29, 30). Site-specific *N-*glycan analysis of FcγRIIIa from different sources shows extensive compositional heterogeneity ranging from oligomannose structures to complex sialylated tetra-antennary glycans with LacNAc extensions. These site-specific differences in *N-*glycan processing depend on cell type and individual (31–36). Similar to the unique restricted *N-*glycan processing seen for IgG-Fc Asn297, the Asn162 and Asn45 in FcγRIIIa also appear to exhibit restricted *N-*glycans processing. Furthermore, FcγRIIIa expressed on NK cells shows the highest level of oligomanosylated Asn162 and Asn45, followed by FcγRIIIa on monocytes and finally recombinantly expressed FcγRIIIa (37). Recombinantly expressed FcγRIIIa obtains almost exclusively complex-type *N-*glycans that correlate with the repertoire of glycosyltransferases expressed in these respective cell lines (37).

While the influence of glycosylation of IgG is well studied, few studies have addressed the influence of FcγRIIIa *N-*glycans on IgG binding and subsequent immune cell activation. Several limitations complicate FcγRIIIa glycan studies, such as the number of *N-*glycan sites with their inherent heterogeneity, the scarcity of endogenous FcγRIIIa material, and the fact that recombinantly expressed FcγRIIIa in HEK293 (33, 38), CHO (33, 39), NS0 (40), and BHK (41) appears to have *N-*glycan structures dissimilar to those found on endogenously expressed FcγRIIIa (32, 37). However, recent studies of IgG : FcγRIIIa interactions revealed that FcγRIIIa with an oligomannose structure at Asn162 has increased binding capacity to IgG1 (35, 42, 43). Furthermore, FcγRIIIa with hybrid-type and truncated *N-*glycan, which existed of only the innermost GlcNAc at Asn162, also has an increased IgG1 affinity, whereas sialic acid (Sia) had a negative influence on IgG1 binding and was shown to induce increased dissociation (32, 38, 43, 44). Furthermore, several limitations, such as the experimental surface plasmon resonance (SPR) setup, antibody and FcγR allotype and glycosylation heterogeneity, make comparing affinity measurements across studies challenging. Due to the recent wide availability of gene editing, the heterogeneity of these *N-*glycans can now be limited and defined glycan structures of interest can be expressed on IgG and Fc Receptors, among many other glycoproteins (45–47).

Here, we aimed to systematically study the impact of *N-*glycosylation features on both IgG1 and FcγRIIIa in their interaction. For this, we produced a library of defined glycoengineered IgG1 and FcγRIIIa (158F and 158V allotypes) glycoproteins and tested these in binding by SPR. The results show that the interaction between afucosylated IgG and FcγRIIIa with oligomannose *N-*glycans produces the highest achievable affinity in our setup. Our study supports previous findings (32, 38, 43, 44) and demonstrates that *N-*glycosylation of not only IgG but also FcγRIIIa affects binding interactions. Furthermore, FcγR glycosylation studies in both health and disease should be performed to discover how the immune system regulates this glycosylation interplay to drive pro- or anti-inflammatory responses.

## Results

### Glycoengineering and production of recombinant IgG1 glycoforms

**Figure 1:**
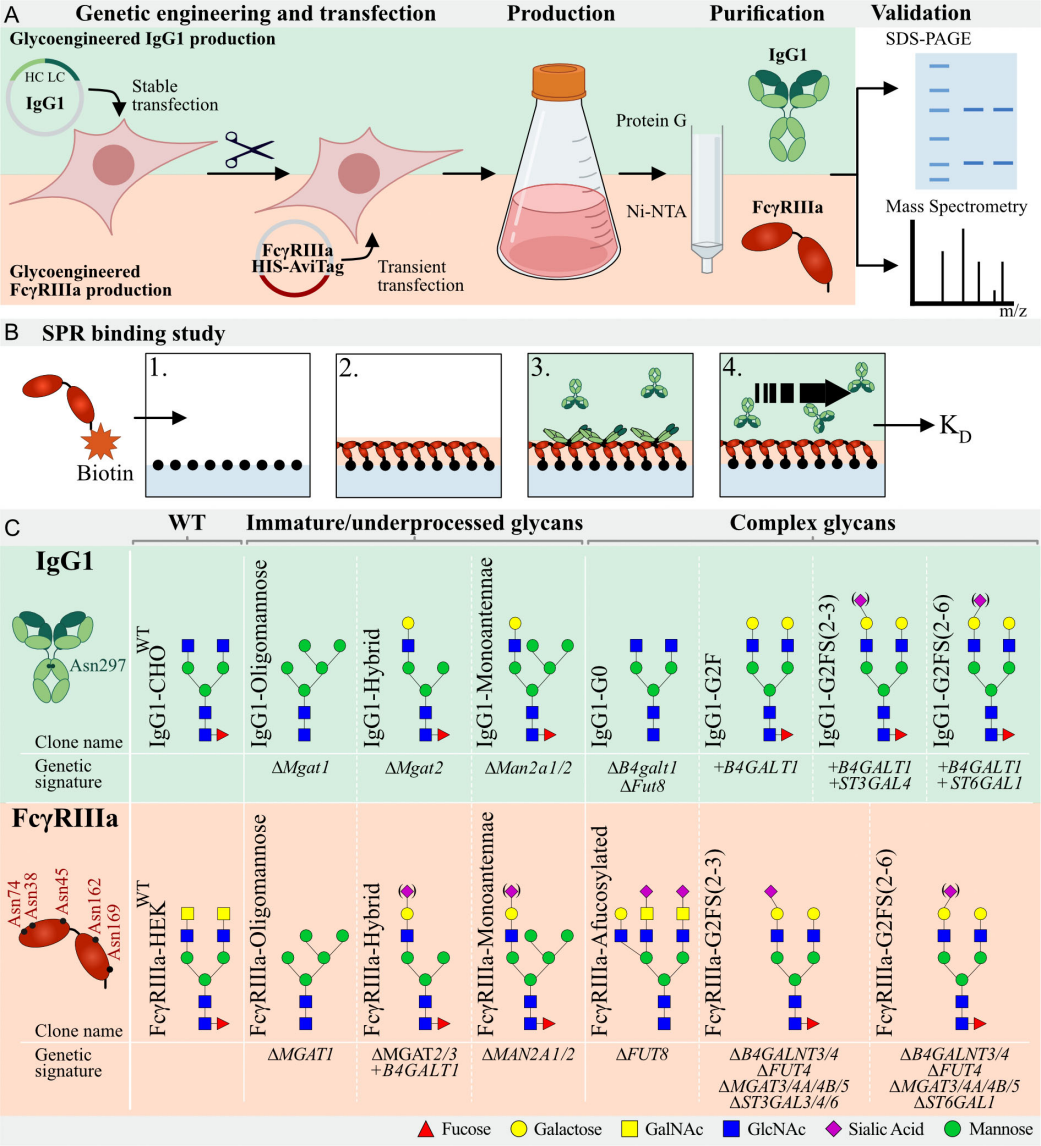
Graphic depiction of glycoengineered IgG1 and FcγRIIIa production, validation, and binding studies. **(A)** Top: Production of CHO^WT^ stably expressing IgG1. In this parental clone, KO and KI of glycosyltransferases resulted in a library of genetically glycoengineered CHO clones stably producing IgG1. Expressed IgG1 was purified by protein G and validated by SDS-PAGE and Mass Spectrometry (MS). Bottom, HEK293 cells were genetically engineered to display distinct *N-*glycan structures and this library of genetically engineered HEK clones were transiently transfected with soluble HIS- and AviTag-tagged FcγRIIIa-158F/V. Produced FcγRIIIa was purified by Ni-NTA columns and subjected to SDS-PAGE and MS for validation. **(B)** Schematic depiction of the IBIS MX96 SPR setup. 1) All glycoengineered FcγRIIIa glycoforms were enzymatically biotinylated by BirA and spotted at four different concentrations on a streptavidin-coated chip. 2) Glycoengineered IgG1 was injected at eight different dilutions, 3) allowing for binding affinity measurements of each antibody to all glycoengineered FcγRIIIa’s simultaneously. 4) Regeneration after every sample was carried out after which the next IgG1 glycoform was injected. **(C)** Glycoengineered IgG1 and FcγRIIIa-158F/V expressing cell lines with clone name, gene editing background and expected *N-*glycan signature based on gene editing signature and literature. IgG1 has one *N-*glycan site (Asn297) per Fc domain and FcγRIIIa has 5 *N-*glycan sites (Asn38, Asn45, Asn74, Asn162, and Asn169). Designations for monosaccharides are according to the Consortium for Functional Glycomics (CFG) (51).

A panel of glycoengineered IgG1 (mAb SO57) (48, 49) with different *N-*glycoforms using stably glycoengineered CHO cells was previously generated (45). In brief, we first established a CHO wildtype clone stably expressing IgG1 (IgG1-CHO^WT^) and used IgG1-CHO^WT^ to generate a library of *N-*glycoengineered clones by knockout (KO) using CRISPR/Cas9 and stable knock-in (KI) of key glycosyltransferases in the *N-*glycosylation pathway using Zinc finger nucleases and the ObLiGaRe Strategy (50) (**Figure 1**, **Supplementary Table S1**). To obtain afucosylated IgG1, a *Fut8* and *B4galt1* KO was created, resulting in the IgG1-G0 clone. An *Mgat1* KO was created (IgG1-Oligomannose) to obtain oligomannosylated IgG1 and KO of *Mgat2* and *Man2a1/2* resulted in hybrid (IgG1-Hybrid) and monoantennary (IgG1-Monoantennae) IgG1-expressing clone, respectively. To increase galactosylation compared to IgG1-CHO^WT^, the IgG1-G2F clone was created by KI of *B4GALT1*. To further increase linkage-specific sialylated IgG1, KI of *ST3GAL4* or *ST6GAL1* was introduced on top of the *B4GALT1* KI clone, resulting in α2-3 linked (IgG1-G2FS(2-3)) or α2-6 linked Sia (IgG1-G2FS(2-6)), respectively, as previously described (45) (**Figure 1**, **Supplementary Table S1**).

**Figure 2:**
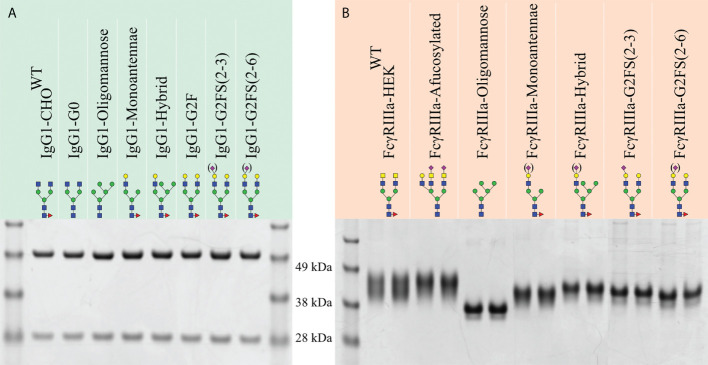
SDS-PAGE of purified recombinant IgG1 and FcγRIIIa-158F/V. Reducing SDS-PAGE of 1 μg purified **(A)** glycoengineered IgG1 produced in CHO cells with the heavy chain and light chain at ~50 and 30 kDa, respectively. **(B)** HEK293-expressed His- and AviTag-tagged FcγRIIIa-158F (left lane) and -158V (right lane) migrating as a broad band with a molecular weight ranging from 40 to 50 kDa. Designations for monosaccharides according to the CFG are indicated (51).

The isolated glycoengineered IgG1 glycoforms were validated by SDS-PAGE for correct protein expression and purity, and to confirm concentration determination. All IgG1 glycoforms resulted in a heavy chain (HC) and low chain (LC) band of 50 and 25 kDa, respectively (**Figure 2A**). Further validation of glycosylation patterns was done by MALDI-TOF-MS employing linkage-specific sialic acid derivatization to differentiate between α2-3 and α2-6 linked Sia (**Supplementary Figure S1**) **(** 52, 53). IgG1-CHO^WT^ showed a heterogeneous profile including dominant G0F glycans with small amounts of G1F (**Supplementary Figure S1A**) (45). Homogenous afucosylated, agalactosylated IgG1 was produced by IgG1-G0 (**Supplementary Figure S1B**) and homogenous Man5 IgG1 by IgG1-Oligomannose (**Supplementary Figure S1C**). The dominant glycoform for IgG1-Monoantennae was the galactosylated monoantennary IgG1 with small amounts being agalactosylated and minor amounts being galactosylated and α2-3 sialylated (**Supplementary Figure S1D**). The same galactosylation and sialylation trend was seen for IgG1-Hybrid (**Supplementary Figure S1E**). IgG1-G2F produced homogenous G2F, which is a significant galactosylation increase in comparison to IgG1-CHO^WT^ (**Supplementary Figure S1F**). For both sialyltransferase KI clones (IgG1-G2FS(2-3) and IgG1-G2FS(2-6)), the dominant glycoforms remained G2F, with a minority of species being sialylated (**Supplementary Figures S1G, H**). Sialylation in IgG1-G2FS(2-6) was lower compared to our previous study (45), and this may be due to differences in cell density and viability during cell culturing. However, sialylation was increased compared to IgG1-CHO^WT^ and IgG1-G2F (**Supplementary Figures S1A, F–H**).

### Glycoengineering and production of recombinant FcγRIIIa-158F/V glycoforms

FcγRIIIa has five consensus *N-*glycosites occupied by heterogeneous *N-*glycans. These display site-specific *N-*glycan structures and variation hereof in different cell types and individuals (54). We generated a library of glycoengineered HEK293 cells with different *N-*glycosylation capacities by combinatorial KO and KI of glycosyltransferase genes as previously reported (47). For afucosylated FcγRIIIa, *FUT8* was knocked out. To obtain oligomannose, monoantennae, and hybrid FcγRIIIa-158F/V we used KO of *MGAT1*, *MGAT2/3*, or *MAN2A1/2*, respectively. KO of *B4GALN3/4* and *MGAT3/4A/4B/5* in combination with *ST3GAL3/4/6* or *ST6GAL1*, resulted in clones expressing complex-type biantennary α2-3 or α2-6 linked sialylated *N-*glycans without LacNAc repeats, respectively. Recombinant FcγRIIIa-158F and 158V were transiently expressed in CHO^WT^ and HEK293^WT^, resulting in FcγRIIIa-CHO^WT^ and FcγRIIIa- HEK^WT^, respectively (**Figure 1**, **Supplementary Figure S2**, **Supplementary Table S1**). Transfection of the above-mentioned glycoengineered HEK cells resulted in FcγRIIIa-Afucosylated, FcγRIIIa-Hybrid, FcγRIIIa-Oligomannose, FcγRIIIa-Monoantennae, FcγRIIIa-G2FS(2-3), and FcγRIIIa-G2FS(2-6) (**Figure 1**, **Supplementary Figure S2**, **Supplementary Table S1**).

**Figure 3:**
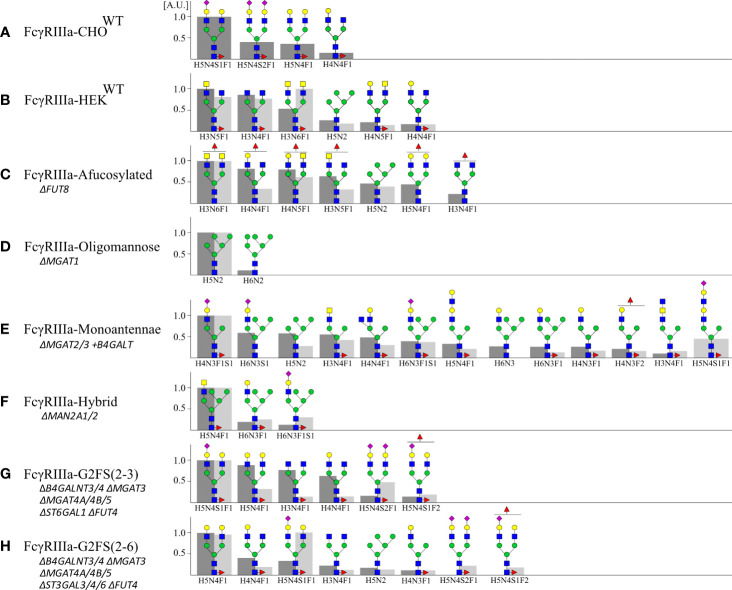
Site-specific *N-*glycan profiling of Asn162 FcγRIIIa-158F/V by LC-MS/MS. FcγRIIIa-158F and -158V expressed in **(A)** CHO and **(B–H)** HEK293 cells were digested by a combination of chymotrypsin and Glu-C and subjected to LC-MS/MS. Site-specific *N-*glycan profiling of Asn162 was carried out and the top 90% of the structures are represented relative to the most abundant structure (A.U. = 1) for FcγRIIIa-158F (dark grey, left) and -158V (light grey, right) with H: hexose, N: N-acetylhexosamine, F: fucose; S: Sialic acid. Proposed glycan structures are based on gene editing signatures and literature. Structures are depicted following the CFG notation (51).

The produced FcγRIIIa-158F/V were validated by SDS-PAGE and mass spectrometry to confirm *N-*glycan structures and evaluate glycan heterogeneity (**Figures 2**, **3**, **Supplementary Figures S3, 4**). Glycoengineered FcγRIIIa resulted in a heterogeneous glycoprofile migrating as a broad band with an apparent molecular weight ranging from 40 to 50 kDa depending on clone, highlighting the *N-*glycan heterogeneity and mass contribution of the five *N-*glycans per FcγRIIIa molecule.

For glycoprofiling, PNGaseF-released esterified *N-*glycans of FcγRIIIa-158F/V were analyzed by MALDI-TOF-MS (**Supplementary Figures S3, 4**) **(** 52). Since the Asn162 glycosite of FcγRIIIa has shown to influence IgG1 binding (29), site-specific analysis of Asn162 by LC-MS/MS was performed. For this, FcγRIIIa was digested by chymotrypsin and Glu-C before subjection to LC-MS/MS and the top 90% most abundant glycan structures for the Asn162 glycosite are depicted (**Figure 3**). In general, the Asn162 *N-*glycan structures for both FcγRIIIa-158F/V confirm gene editing signatures and generally followed MALDI-TOF-MS data. However, underprocessed glycans were seen for Asn162 in comparison to the total *N-*glycan profiling, where more complex *N-*glycans were found for FcγRIIIa-HEK^WT^, FcγRIIIa-CHO^WT^, and FcγRIIIa-afucosylated (33, 35). The Asn162 of FcγRIIIa-CHO^WT^ was predominantly of the G2S type with overall high levels of galactosylation (G2S>G2S2>G2) and variable sialylation levels (G2S>G2S2) (**Figure 3**, **Supplementary Figures S3A, 4A**). The FcγRIIIa-HEK^WT^ Asn162 site had mainly biantennary *N-*glycans with GalNAc extensions and no sialylation. These were also the major glycan species found for the total FcγRIIIa-HEK^WT^ glycoprofiling data (**Figure 3B**). *N-*glycan profiling of FcγRIIIa-HEK^WT^ also showed minor sialylated, elongated, and branched glycospecies (**Supplementary Figures S3A, 4A**). Most complex *N-*glycans were seen for FcγRIIIa-afucosylated, supporting the higher molecular weight as seen on SDS-PAGE (**Figures 2B**, **3C**, **Supplementary Figures S3B, 4B**). Confirmed by total protein glycoprofiling and Asn162 site-specific glycoanalysis, homogeneous Man5 was found for FcγRIIIa-Oligomannose (**Figure 3D**, **Supplementary Figures S3C, 4C**). The major Asn162 glycan structure for FcγRIIIa-Monoantennae is a sialylated monoantennary species **(** **Figure 3E**, **Supplementary Figures S3D, 4D** **)**. However, a wider variety of other glycan structures were seen for both Asn162 and total FcγRIIIa glycoprofiling such as hybrid species and biantennary *N-*glycans and total *N-*glycoprofiling of FcγRIIIa-hybrid clone showed more complex N-glycans than expected. For both FcγRIIIa-G2FS(2-3) and FcγRIIIa-G2FS(2-6), biantennary *N-*glycans with variable sialylation levels were seen (**Figures 3G, H**, **Supplementary Figures S3F, G, 4F, G**). Furthermore, branching fucose was found in FcγRIIIa-G2FS(2-3) and -G2FS(2-6).

### *N-*glycan structures on both IgG1 and FcγRIIIa-158F/V influence their binding interaction

To study the role of *N-*glycoforms on the IgG1:FcγRIIIa interaction, we used the produced glycovariants for SPR analysis on the IBIS MX96 biosensor system, as described in Dekkers et al. (55). For this all FcγRIIIa-158F/V glycoforms were enzymatically biotinylated at the AviTag™ and immobilized on a sensor chip. Subsequently, all IgG1 variants were injected as analytes in a multi-cycle mode (**Figure 1B**). Humira, a recombinant human IgG1 monoclonal antibody with a G0F or G0NF glycan structure (56), and recombinant FcγRIIIa-158F/V purchased from SinoBiologicals, were used as controls (**Supplementary Table S2**). Binding was observed for all IgG1:FcγRIIIa combinations, however, the FcγRIIIa-158F binding to IgG1-Monoantennae and IgG1-Hybrid was weak and therefore K_D_ values could not be calculated from these interactions (**Supplementary Figures S5, 2**).

**Figure 4:**
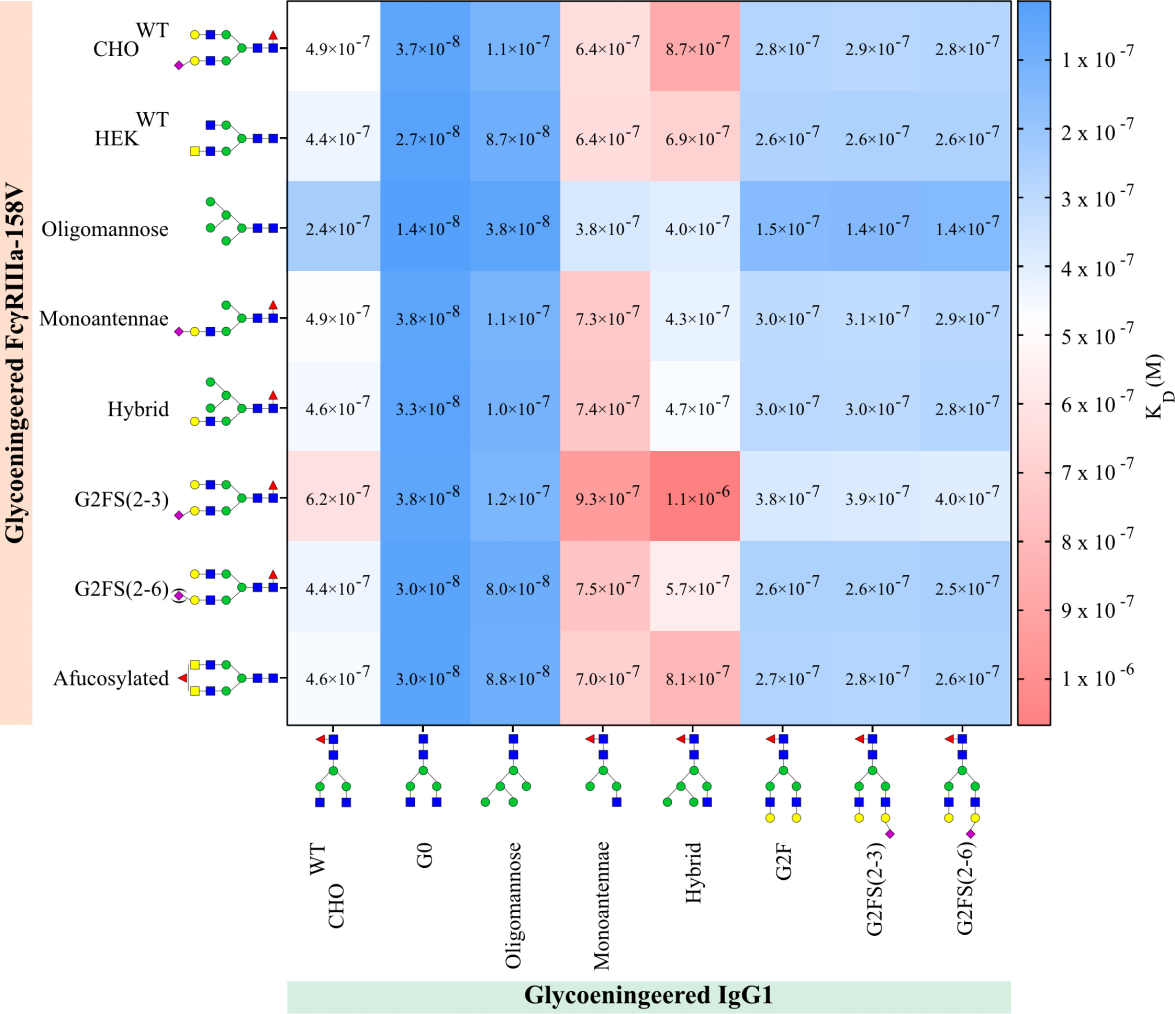
IgG1 and FcγRIIIa-158V binding. Surface plasmon resonance dissociation constant (K_D_) was determined from SPR analysis after biotinylated glycoengineered FcγRIIIa-158V was spotted at 4 concentrations and bound to glycoengineered IgG1 at 8 times dilution series. Calculation of the dissociation constant was performed by equilibrium fitting to R_max_= 500. Mean data is reported of three independent experiments for each IgG1:FcγRIIIa-158V pair.

We could confirm that FcγRIIIa-158V exhibited on average a 3-6 fold increased affinity for IgG1 compared to FcγRIIIa-158F regardless of the IgG1 glycoform tested (**Figure 4**, **Supplementary Table S2**). The highest affinity for all IgG1 glycoforms was seen to FcγRIIIa-Oligomannose with a 2-3 fold increase compared to all other FcγRIIIa glycoforms (**Figure 4**, **Supplementary Table S2**). Surprisingly, FcγRIIIa-Monoantennae and FcγRIIIa-Hybrid, carrying immature *N-*glycan structures often found on Asn162 expressed by native immune cells (57), had a similar affinity to all IgG1 glycoforms when compared to FcγRIIIa-HEK^WT^ and FcγRIIIa-CHO^WT^, with the exception of IgG1-hybrid (**Figure 4**, **Supplementary Table S2**). Sialylation of FcγRIIIa (FcγRIIIa-G2FS(2-3) and FcγRIIIa-G2FS(2-6)) influenced IgG1 binding to a certain degree, with FcγRIIIa-2FS(2-3) having approximately a 2-fold decrease in binding compared to FcγRIIIa-G2FS(2-6) and FcγRIIIa-HEK^WT^. However, it needs to be taken into account that the sialylation levels for FcγRIIIa-G2FS(2-3) are much higher compared to G2FS(2-6), where G2F is the dominant glycoform on Asn162 (**Supplementary Figures S3F, G**). Surprisingly, FcγRIIIa-G2FS(2-3) has a lower affinity to most glycoengineered IgG1 compared to FcγRIIIa-CHO^WT^, even though the Asn162 glycan pattern is similar.

As expected, the highest affinity of all FcγRIIIa receptors was observed to afucosylated IgG, both IgG1-G0 and the IgG1-Oligomannose (**Figure 4**, **Supplementary Table S2**) **(** 29). Interestingly, the *N-*glycosylation state of the FcγRIIIa had minimal effect on the binding affinity when probed with afucosylated IgG, except for oligomannosylated FcγRIIIa where binding affinity is increased by a factor of two. All FcγRIIIa glycoforms bound equally to IgG1 capped by α2-3 or α2-6 Sia (IgG1-G2FS(2-3) and IgG1-G2FS(2-6)), with minor differences that appear to be negligible (**Figure 4**, **Supplementary Table S2**). The highest K_D_, and thus lowest affinity, was seen for IgG1-Hybrid and IgG1-Monoantennae to all FcγRIIIa. In contrast to IgG1-F0, for these suboptimal glycoengineered IgG1, the FcγRIIIa glycosylation influences binding (**Figure 4**, **Supplementary Table S2**). This combinatorial setup with glycoengineered IgG1 and FcγRIIIa-158F/V confirms that afucosylated IgG1 has the highest binding affinity to oligomannosylated FcγRIIIa-158V (FcγRIIIa-Oligomannose).

## Discussion

Here, we systemically produced a library of defined *N-*glycoforms of FcγRIIIa (both 158F/V allotypes) and IgG1 to study *N-*glycosylation binding features and used combinations of these glycoforms with SPR to evaluate binding kinetics. Our study demonstrated that rather homogenous *N-*glycoforms of FcγRIIIa and IgG1 can be produced in genetically glycoengineered CHO and HEK293 mammalian cells as previously reported (45, 49). Importantly, the genetic engineering approach does not enable control of *N-*glycan structures at specific sites, but rather sets global restrictions on the repertoire of *N-*glycan structures on a given glycoprotein. Thus, the genetic engineering design will apply to all *N-*glycosites, except for those sites where *N-*glycan processing is naturally constrained by interaction with the protein backbone and glycosyltransferase substrate specificity, as for the Asn297 *N-*glycan of IgG1 (4). An IgG consists of two heavy chains, identical on the protein level, each having one *N-*glycosite, implying that one IgG molecule carries two glycan structures. The glycan composition of these two glycans might be different, referred to as an asymmetrically glycosylated antibody. Here, the obtained K_D_ measurements are an average of the IgG glycoforms population to a given population of FcγRIIIa glycoforms. However, as the antibody and receptor in this study are produced in the same cell with the same constrained glycoengineering background, we believe differences in glycan composition between these two glycans are minimal, and IgG1-Oligomannose, IgG1-G0 and IgG1-G2F are symmetrically glycosylated (45). While this conserved Fc *N-*glycan is unique by its restricted branching, galactosylation, and sialylation, many other *N-*glycoproteins are also known to have specific differentially processed *N-*glycosites, such as an oligomannose *N-*glycan on IgM or Man-6-phosphate tagging of lysosomal enzymes (46, 58–60). The Asn162 glycosite in FcγRIIIa and the adjacent 158F/V polymorphism are also interesting in this aspect, as this site is often occupied by oligomannose, monoantennae, and hybrid *N-*glycans in human leukocytes (31–34).

In our setup, a remarkable >300-fold difference in binding affinities was observed among the different IgG1 glycoforms and FcγRIIIa allotype and glycoforms tested. It is however important to note that these results are based on *in vitro* binding K_D_ values and may not fully translate into the *in vivo* properties of these interactions. *In vivo*, monovalent IgG competes with multivalent IgG-immunocomplexes or IgG-opsonized pathogens for FcR binding, of which only the latter results in FcγR-mediated cellular activation. Nevertheless, the reported relative binding affinities between FcγRIIIa and IgG1 are known to be reproducibly translated into relative differences in cellular effector functions as well as *in vivo* functional capacities (8, 24, 61). How these affinity changes translate into different activation potentials of NK cells or myeloid expressing cells, depends on many factors, such as epitope density of the target cell, antibody concentration, FcγR-polymorphisms of the patient/donor cells, and the antibody Fc-glycosylation, especially fucosylation, which can be highly variable in some immune response (11). These factors all impact avidity between the target and effector cell, which seems the principle component governing the FcγR-activation potential (24). Our results now suggest that FcγRIII-glycosylation itself can impact this as well, which has been reported to be differently glycosylated across cell types.

The FcγRIIIa-158V allotype has a higher binding affinity to IgG1 compared to the 158F allotype (62). This has recently been attributed to the formation of a less stable complex between the 158F allotype with IgG in comparison to the I58V allotype (63). This is also confirmed in our setup, where the FcγRIIIa-158V binding affinity is on average 4-6 times higher compared to FcγRIIIa-158F. Besides the different allelic variants, the FcγRIIIa *N-*glycosylation of both the 158F and V allotype also influences IgG1 binding (29, 30, 63). The Asn162 *N-*glycan site, and to a lesser extent Asn45, have shown to affect antibody-binding affinity (29, 30). For the remaining *N-*glycosites, specific functions remain unclear. Our findings showed that, even though all FcγRIIIa N-glycan forms are capable of binding IgG and subsequently activate the immune system, specific glycoforms are more capable of binding IgG. This is reflected by the 43- and 76-fold binding difference for the highest and lowest binding FcγRIIIa glycoforms for FcγRIIIa-158F and -158V, respectively (32). Interestingly, these stronger-binding oligomannose, hybrid, and monoantennae *N-*glycans, shown to have ~2.5-fold improved IgG1 binding compared to complex-type N-glycans, are often found on native FcγRIIIa expressed on human leukocytes (31–35, 37, 42, 43).

In this setup, the highest affinity to IgG1 was indeed seen for FcγRIIIa-Oligomannose, which was on average 2-3 fold better compared to all other FcγRIIIa glycoforms, in agreement with recent reports (35, 43). The FcγRIIIa-Oligomannose inherently also lacks the core fucose as the FUT8 α1-6 fucosyltransferase requires complex *N-*glycans as acceptor substrate (64). We could exclude that FcγRIIIa core fucose was solely responsible for the increased affinity to IgG1, since FcγRIIIa-Afucosylated did not have an increased binding affinity to IgG1 when compared to the fucosylated complex-type FcγRIIIa. Interestingly, the *N-*glycoforms of FcγRIIIa-Afucosylated tended to have higher levels of complex structures with a higher degree of branching, which may affect the interpretation. Roberts et al. noted a ~2-fold increase for hybrid-type *N-*glycosylated FcγRIIIa (32). In contrast to the literature, in our setup, mono-antennae and hybrid *N-*glycosylated FcγRIIIa showed affinities to IgG1 similar to FcγRIIIa with complex *N-*glycan. The sialylation state of *N-*glycans on FcγRIIIa appeared to influence the IgG1 binding affinity with a decrease in binding affinity of α2-3 Sia capping compared to α2-6 Sia. Unfortunately, it was not possible to substantially increase sialylation, which is a well-known issue for IgG (45, 65–67). Furthermore, due to the differences in sialylation levels between the 158F and V allotype at the Asn162 glycosite, the comparison to asialylated FcγRIIIa is challenging and this conclusion should be treated with caution.

Our studies demonstrated that the combination of oligomannosylated FcγRIIIa had the highest binding affinity to afucosylated IgG1, which is in agreement with previous reports (6–8, 35, 43). In general, the FcγRIIIa *N-*glycosylation state had minimal influence on the binding affinity when probed with afucosylated IgG1. However, when FcγRIIIa was probed with IgG1 fucosylated glycoforms, the FcγRIIIa *N-*glycosylation state influenced the IgG1 binding affinity. Furthermore, IgG1 sialylation had a minimal effect on FcγRIIIa affinity, and differences in α2-3- and α2-6-linked Sia were negligible, as shown before (8, 68, 69). This, however, is in contrast with findings from other groups, which could be due to differences in sialylation levels (8, 70, 71). These findings likely translate into differential sensitivity of effector cells with its inherent FcγRIIIa glycosylation state to the IgG1 fucosylation state. A higher discriminatory power for the fucosylation state of IgG1 by oligomannosylated FcγRIIIa (~17-fold) was found, a glycan structure typically found on NK cells. For the hybrid and mono-antennary FcγRIIIa glycoforms, typically found on monocytes, this discrimination between fucosylation states of IgG1 was slightly less, or a ~13-fold increased preference for afucosylated IgG1. It remains unknown to which extent differential FcγRIII glycoprocessing on different cell types affects their activation state and future studies should address the biosynthetic and mechanistic basis for differential site-specific *N-*glycosylation of FcγRIIIa.

In summary, our study demonstrates that the *N-*glycosylation status of both FcγRIIIa and IgG1 influences binding affinity, highlighting the need to consider the *N-*glycosylation state of both IgG and Fc receptors in binding kinetics and effector function studies. The two most significant *N-*glycosylation features observed were the IgG1 core fucosylation state and FcγRIIIa oligomannose to complex *N-*glycosylation transition. We predict that the bilateral glycan interplay enables the immune system to fine-tune the FcγRIIIa and IgG interactions and modulate immune responses.

## Methods

### Cell culture

HEK293 were grown in suspension in serum-free F17 culture media (Invitrogen) supplemented with 0.1% Kolliphor P188 (SIGMA) and 4 mM GlutaMax as previously described (47). CHOZN GS-/- cells (Sigma) were maintained as suspension cultures in serum-free media (EX-CELL CHO CD Fusion). Cultures were grown at 37°C and 5% CO_2_. HEK293 and CHO cultures were under constant agitation (120 rpm).

### CRISPR/Cas9 targeted KO in HEK293 cells

KO was performed using a validated gRNA library for all human glycosyltransferases (GlycoCRISPR) (72), as previously described (73). In brief, HEK293 cells were seeded in 6-well plates (NUNC), transfected with 1 μg of gRNA and 1 μg of GFP-tagged Cas9-PBKS using Lipofectamine 3000 (Invitrogen) according to manufacturer’s instructions. Cells were harvested after 24 hours, and bulk sorted for GFP expression by FACS (SONY SH800). After culturing, the sorted cell pool was further single-sorted into 96 well plates and screened by Indel Detection by Amplicon Analysis (IDAA) (74), and indels of selected clones were confirmed by Sanger sequencing.

### CRISPR/Cas9-targeted KO in CHO cells

One day prior to transfection, cells were seeded in T25 flasks (NUNC). Electroporation was conducted with 2 × 10^6^ cells with 1 μg of both endotoxin-free plasmid DNA of Cas9-GFP and gRNA in the U6GRNA plasmid (Addgene Plasmid #68370) using Amaxa Kit V and program U-24 with Amaxa Nucleofector 2B (Lonza). Electroporated cells were moved to a 6-well plate with 3 ml growth media. Forty-eight hours after nucleofection, the 10–15% highest GFP-labeled pool of cells was enriched by FACS, and after one week of culturing, cells were single-cell sorted by FACS (SONY SH800) into 96-well plates. KO clones were identified by IDAA, as mentioned above, as well as immunocytology with appropriate lectins or monoclonal antibodies, whenever possible. Selected clones were further verified by Sanger sequencing.

### ZFN/CRISPR-mediated KI in CHO cells

Site-specific CHO Safe-Harbor locus KI was based on ObLiGaRe strategy (50) and performed with 2 µg of each ZFN (Merck/Sigma-Aldrich) tagged with GFP/Crimson (74), and 5 µg donor plasmid with full coding human genes, as described before (46).

### IgG1 expression and purification

The anti-rabies human IgG1 SO57 (48) was used to establish stable expressing CHO clones as described by Schulz et al. (45). For IgG production, clones were seeded at 0.5 x 10^6^ cells/ml without L-glutamine and cultured for three days. IgG was purified by HiTrap™ protein HP (CE Healthcare) as previously described (45). Protein purity and concentration were evaluated by SDS-PAGE and Coomassie staining.

### Transient expression of soluble FcγRIIIa in HEK cells and protein purification

HEK293-6E cells were seeded at a cell density of 0.5 × 10^6^ cells/ml and transfected with FcγRIIIa-158F/V with 10X his-tag and AviTag™ the next day with 1:3 μg DNA : PEI per 1 × 10^6^ cells. Secreted protein was harvested after 72 hours and purified from culture medium by nickel affinity chromatography. For this, the culture medium was centrifuged, filtered (0.45 μm), mixed 3:1 (v/v) in 4X binding buffer (100 mM sodium phosphate, pH 7.4, 2 M NaCl) and applied to self-packed nickel-nitrilotriacetic acid (Ni-NTA) affinity resin column (Qiagen), which was pre-equilibrated in washing buffer (25 mM sodium phosphate, pH 7.4, 500 mM NaCl, 20 mM imidazole). After washing, bound protein was eluted with 250 mM imidazole in washing buffer. Purity and quantification were evaluated by SDS-PAGE and Coomassie staining. Purified protein was buffer-exchanged to approximately 1 mg/ml in 50mM AmBic buffer with 2 ml Zeba Spin Desalting Column 7K MWCO (ThermoFisher).

### Stable expression of soluble FcγRIIIa in CHO cells and protein purification

Both FcγRIIIa-158F/V with 10X his-tag and AviTag™ constructs were both subcloned into a modified pCGS3 vector (Merck/Sigma-Aldrich) for glutamine selection in CHOZN GS−/− cells (Sigma). CHO cells were seeded at 0.5 X 10^6^ cells/ml in T25 flasks (NUNC) one day prior to transfection. Two million cells were electroporated with 5 μg plasmids using Amaxa kit V and program U24 with Amaxa Nucleofector 2B (Lonza) and plated in 6-wells with 3 ml growth media. Three days after transfection, cells were plated in 96-wells at 1000 cells/well in 200 μl Minipool Plating Medium containing 80% EX-CELL^®^ CHO Cloning Medium and EX-CELL CHO CD Fusion serum-free media without glutamine (Sigmaaldrich). High-expressing clones were selected by testing the medium using anti-HIS antibodies, and selected clones were scaled up in serum-free media without L-glutamine TPP TubeSpin^®^ shaking Bioreactors (180 rpm, 37 °C, and 5% CO_2_) for protein production. FcγRIIIa was purified as described above.

### *N-*glycan profiling by MALDI-TOF

*N-*glycan protein profiling employing linkage-specific sialic acid esterification was obtained as described by Reiding et al. (52). In brief, 10 μg IgG1 and FcγRIIIa were denatured by incubating the samples 10 min at 60°C in 2% SDS. *N-*glycans were released by adding a release mixture containing 2% NP-40 and 0.5 mU PNGaseF at 37°C over night. Released *N-*glycans were esterified to obtain sialic acid linkage specificity. In brief, esterification reagent containing 0.5 M EDC and 0.5 M HOBt in ethanol were added to 1 μl glycan mixture and incubated for one hour at 37°C. After incubation, 25% NH_4_OH and subsequently 100% ACN was added (52). Glycan enrichment was performed by cotton hydophilic interaction liquid chromatography (HILIC) (75). Briefly, pipet tips were packed with cotton thread which was conditioned by 85% ACN. The sample was loaded by pipetting 20 times into the reaction mixture. Tips were washed in 85% ACN 1% TFA, followed by 85% ACN and eluted in 10 μl MQ. For MALDI-TOF-MS analysis, 1μl HILIC purified glycan sample was spotted on an MTP AnchorChip™ 384 BC mixed on plate with 1μl sDHB (5mg/ml in 50% ACN) and left to air dry. The sample was recrystallized using 0.2μl ethanol. Measurement was performed on the Bruker Autoflex (Bruker Daltonik GmbH, Bremen, Germany) using the Bruker Flex Control 3.4 software. For each spectrum, 10 000 laser shots were accumulated at a laser frequency of 100 Hz. Spectra were recorded in the positive reflector mode (900-4500 Da) and the raw spectra were processed by the Flexanalysis 5.1.

### Sample preparation for site-specific *N-*glycopeptide analysis

The purified protein was dissolved in 50mM ammonium bicarbonate buffer, reduced in 10 mM dithiothreitol (DTT), alkylated with 20 mM iodoacetamide (IAA), reduced again with 10 mM DTT, and digested with 1:20 chymotrypsin:protein followed by 1:20 GluC:protein (Roche). The proteolytic digest was desalted by in-house produced modified StageTip columns containing 3 layers of C18 membrane (3M Empore disks, Sigma Aldrich) (76). Samples were eluted with 50% methanol in 0.1% formic acid (FA), dried down, and re-solubilized in 0.1% FA for LC-MS/MS analysis.

### Site-specific FcγRIIIa *N-*glycopeptide analysis by LC-MS/MS

LC-MS/MS analysis was performed on EASY-nLC 1200 UHPLC (Thermo Scientific) interfaced *via* nanoSpray Flex ion source to an Orbitrap Fusion Lumos MS (Thermo Scientific). Briefly, the nLC was operated in a single analytical column set up using PicoFrit Emitters (New Objectives, 75 μm inner diameter) packed in-house with Reprosil-Pure-AQ C18 phase (Dr. Maisch, 1.9-μm particle size, 19-21 cm column length). Each sample was injected onto the column and eluted in gradients from 3 to 32% B in 75 min, and from 32% to 100% B in 10 min, and 100% B for 10 min at 200 nL/min (Solvent A, 100% H_2_O; Solvent B, 80% acetonitrile; both containing 0.1% (v/v) formic acid). A precursor MS1 scan (m/z 350–2 000) of intact peptides was acquired in the Orbitrap at the nominal resolution setting of 120 000, followed by Orbitrap HCD-MS2 and at the nominal resolution setting of 60 000 of the five most abundant multiply charged precursors in the MS1 spectrum; a minimum MS1 signal threshold of 50 000 was used for triggering data-dependent fragmentation events. Targeted MS/MS analysis was performed by setting up a targeted MS^n^ (tMS^n^) Scan Properties pane. A target list was composed of the top 30 most abundant glycans or glycopeptides from the proposed compositional list. The mass spectrometry proteomics data have been deposited to the ProteomeXchange Consortium *via* the PRIDE partner repository with the dataset identifier PXD035846.

### Data analysis

Glycan and glycopeptide compositional analysis was performed from *m/z* features extracted from LC-MS data using in-house written SysBioWare software, as previously described (46, 77). Briefly, For *m/z* feature recognition from full MS scans LFQ Profiler Node of the Proteome discoverer 2.2 (Thermo Scientific) was used. The list of precursor ions (m/z, charge, peak area) was imported as ASCII data into SysBioWare and compositional assignment within 3 ppm mass tolerance was performed. The main building blocks used for the compositional analysis were: NeuAc, Hex, HexNAc, dHex and the theoretical mass increment of the most prominent peptide corresponding to each potential glycosites. The most prominent peptide sequence related to the *N-*glycosite of interest was determined experimentally by comparing the yield of deamidated peptides before and after PNGase F treatment. One or two phosphate groups were added as building blocks for assignment. To generate the potential glycopeptide list, all the glycoforms with an abundance higher than 10% of the most abundant glycoform were used for glycan feature analysis.

### Surface plasmon resonance

Prior to SPR measurements, glycoengineered FcγRIIIa was site-specifically biotinylated on the BirA tag using BirA enzyme as described by Rodenko et al. (78). For biotinylation of 1 µM FcγRIIIa protein, 3.3 nM BirA ligase was used. After biotinylation overnight at 25°C, the biotinylated FcγRIIIa mixture was buffer-exchanged and subsequently concentrated in PBS pH 7.4 using Amicon Ultra centrifugal filter units (MWCO 3 kDa) (Merck, Millipore).

The biotinylated, recombinant, human FcγRIIIa-158F and 158V from SinoBiological (10389-H27H-B and 10389-H27H1-B, respectively) were used as an control for SPR. SPR measurements were performed on an IBIS MX96 (IBIS technologies) device as described previously by Dekkers *et al.* (8). All biotinylated FcγRIIIa were spotted using a Continuous Flow Microspotter (Wasatch Microfluidics) onto a SensEye G-streptavidin sensor (Senss, 1–08–04–008) allowing for binding affinity measurements of each glycoengineered antibody, and Humira^®^, to all glycoengineered FcγRIIIa simultaneously on the IBIS MX96.

The biotinylated FcγRIIIa were spotted in 4 concentrations with a 3-fold dilution ranging from 0.3 to 100nM, depending on the FcγRIIIa in PBS supplemented with 0.075% Tween-80 (VWR, M126–100ml), pH 7.4. Glycoengineered IgG1 was then injected over the IBIS at 8 times dilution series starting at 15.6 nM until 2000 nM in PBS + 0.075% Tween-80. Regeneration after every sample was carried out with 10 nM Gly-HCl, pH 2.4. Calculation of the dissociation constant (K_D_) was performed by equilibrium fitting to R_max_= 500. Analysis and calculation of all binding data were carried out with Scrubber software version 2 (Biologic Software) and Excel. Three independent experiments for each receptor/Fc pair were carried out on at least two different days and representative data are reported.

## Data availability statement

The data presented in the study are deposited in the ProteomeXchange Consortium via the PRIDE partner repository, accession number PXD035846.

## Author contributions

JVC, HC, and ZY conceived and designed the study. JVC, AB, NdH, MS, LH, ZY, SV, RM, DG and WvE contributed with experimental data and interpretation. JVC, HC, ZY, and GV wrote the manuscript. All authors edited and approved the final version.

## Funding

This work was supported by the Lundbeck Foundation, The Novo Nordisk Foundation, European Commission (GlycoImaging H2020-MSCA-ITN-721297, BioCapture H2020-MSCA-ITN-722171), the Danish National Research Foundation (DNRF107), the National Institutes of Health (AI114730 and R01AI41513, R01AI106987, U01OD024857), Kuang Hua Educational Foundation, The Carlsberg Foundation CF20-0412 and the Landsteiner foundation for Blood Transfusion Research (LSBR) grants 1721 and 1908, and ZonMW COVID-19 grants 1043001 201 0021. Noortje de Haan has received funding from the European Research Council (ERC) under the European Union’s Horizon 2020 research and innovation programme (GlycoSkin H2020-ERC; 772735).

## Conflict of interest

The University of Copenhagen has filed a patent application for the cell-based display platform. GlycoDisplay Aps, Copenhagen, Denmark, has obtained a license in the field of the patent application. Authors YN, ZY, and HC are co-founders of GlycoDisplay Aps and hold ownerships in the company.

The remaining authors declare that the research was conducted in the absence of any commercial or financial relationships that could be construed as a potential conflict of interest.

## Publisher’s note

All claims expressed in this article are solely those of the authors and do not necessarily represent those of their affiliated organizations, or those of the publisher, the editors and the reviewers. Any product that may be evaluated in this article, or claim that may be made by its manufacturer, is not guaranteed or endorsed by the publisher.
