# Supplementary material for: Role of N-Glycosylation in FcγRIIIa interaction with IgG
Source: Front Immunol. 2022 Sep 9;13:987151. doi: 10.3389/fimmu.2022.987151 (PMC9524020; doi:10.3389/fimmu.2022.987151)
Supplement: Supplementary file 2 [file DataSheet_1.pdf]

Role of *N*-glycosylation in FcγRIIIa interaction with IgG

Julie Van Coillie[1,2,3], Morten A. Schulz[1], Arthur E. H. Bentlage[2,3], Noortje de Haan[1], Zilu Ye[1], Dionne M Geerdes[4], Wim J E van Esch[4], Lise Hafkenscheid[1], Rebecca L. Miller[1], Yoshiki Narimatsu[1,5], Sergey Y. Vakhrushev[1], Zhang Yang[1,5,#,*], Gestur Vidarsson[2,3,*] and Henrik Clausen[1,*]

[1] Copenhagen Center for Glycomics, Departments of Cellular and Molecular Medicine, Faculty of Health Sciences, University of Copenhagen, Copenhagen, Denmark;
[2] Department of Experimental Immunohematology, Sanquin Research, Amsterdam, The Netherlands;
[3] Department of Biomolecular Mass Spectrometry and Proteomics, Utrecht Institute for Pharmaceutical Sciences and Bijvoet Center for Biomolecular Research, Utrecht University, Utrecht, The Netherlands;
[4] Sanquin Reagents, Amsterdam, The Netherlands;
[5] GlycoDisplay ApS, Copenhagen, Denmark;
# Present address: Department of Cell Therapy Discovery, Novo Nordisk A/S, Måløv, Denmark;

Supplementary Material

Figure S1. *N*-glycan profiling of IgG1.

Figure S2. FcγRIIIa construct sequence.

Figure S3. *N*-glycan profiling of FcγRIIIa-158F.

Figure S4. *N*-glycan profiling of FcγRIIIa-158V

Figure S5. Sensorgrams of glycoengineered IgG1 and FcγRIIIa-158F/V binding by SPR.

Table S1. Cell line engineering background

Table S2. Binding affinity of glycoengineered IgG1 to FcγRIIIa-158F/V.

Supplementary Figure 1

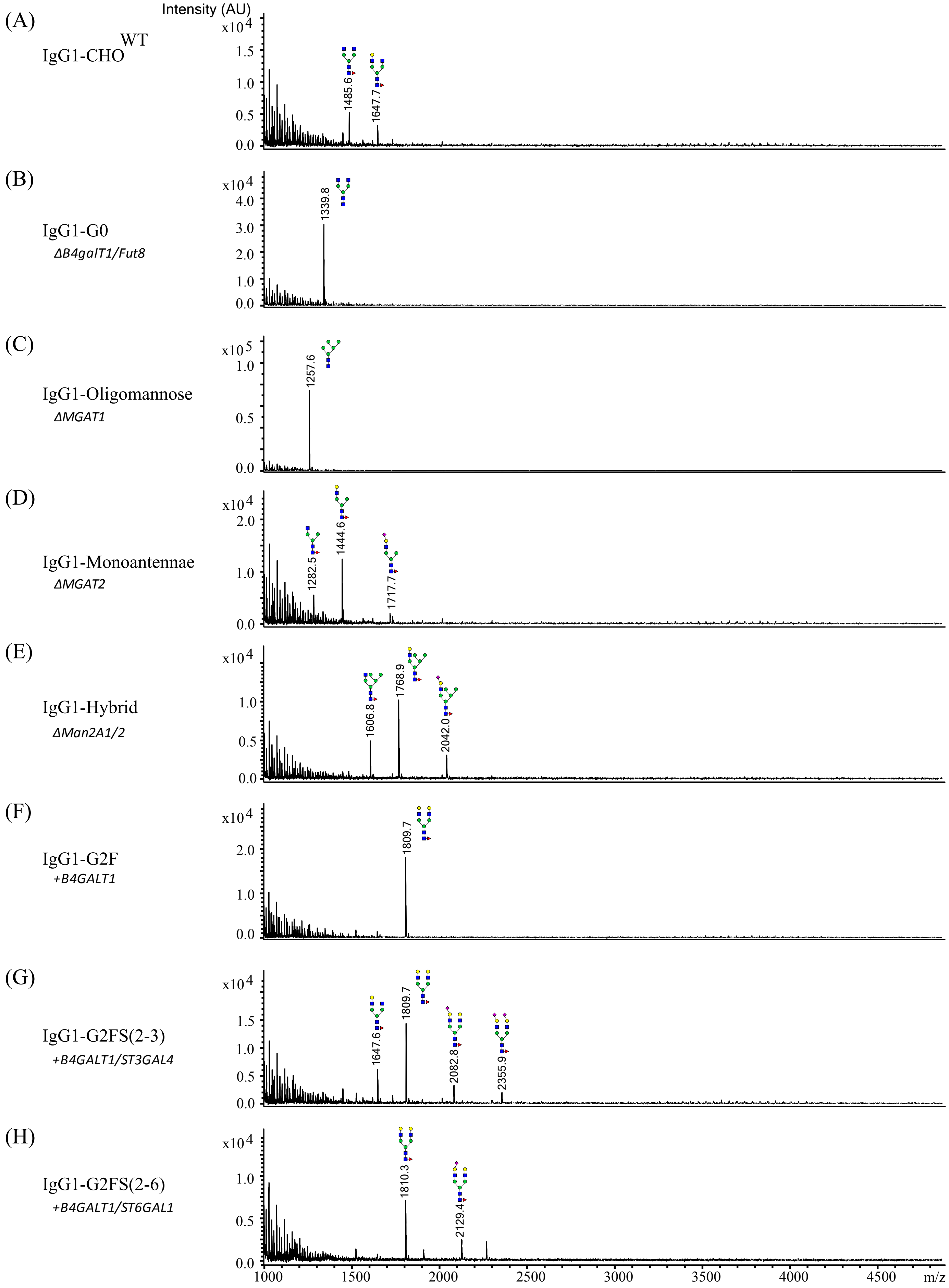

Figure S1. *N*-glycan profiling of IgG1. MALDI-TOF-MS spectrum of esterified released *N*-glycans with m/z range of 1000-5000 according to Reiding *et al.* (52). **(A)** IgG-CHO^{WT}, **(B)** IgG1-G0 (KO of *B4galT1/Fut8*), **(C)** IgG1-oligomannose (KO of *Mgat1*), **(D)** IgG1-Monoantennary (KO of *Mgat2*), **(E)** IgG1-Hybrid (KO of *Man2A1/2*), **(F)** IgG1-G2F (KI of *B4GALT1*), **(G)** IgG1-G2FS(2-3) (KI of *B4GALT1*/*ST3GAL4*), and **(H)** IgG1-G2FS(2-6) (KI of *B4GALT1*/*ST6GAL1*). Annotation represents one possible glycan structure, which is based on gene editing signature and literature. Structures are depicted following the CFG notation (51) and the linkage position of sialic acid is depicted to the left or right for 2-3- or 2-6-linked sialic acid, respectively.

Supplementary Figure 2

```
FcγRIIIA.F  MWQLLLPTAL LLLVSAGMRT EDLPKAVVFL EPQWYRVLEK DSVTLKCQGA
FcγRIIIA.V  MWQLLLPTAL LLLVSAGMRT EDLPKAVVFL EPQWYRVLEK DSVTLKCQGA

                 38        45                              74
FcγRIIIA.F  YSPEDNSTQW FHNESLISSQ ASSYFIDAAT VDDSGEYRCQ TNLSTLSDPV
FcγRIIIA.V  YSPEDNSTQW FHNESLISSQ ASSYFIDAAT VDDSGEYRCQ TNLSTLSDPV

FcγRIIIA.F  QLEVHIGWLL LQAPRWVFKE EDPIHLRCHS WKNTALHKVT YLQNGKGRKY
FcγRIIIA.V  QLEVHIGWLL LQAPRWVFKE EDPIHLRCHS WKNTALHKVT YLQNGKGRKY

                                              162     169
FcγRIIIA.F  FHHNSDFYIP KATLKDSGSY FCRGLFGSKN VSSETVNITI TQEFGGGGSH
FcγRIIIA.V  FHHNSDFYIP KATLKDSGSY FCRGLVGSKN VSSETVNITI TQEFGGGGSH

FcγRIIIA.F  HHHHHHHHHG LNDIFEAQKI EWHE
FcγRIIIA.V  HHHHHHHHHG LNDIFEAQKI EWHE
```

Signal peptide	**K** Biotinylation site
Ig-like domain	**N** N-glycan site
10X HIS-Tag	**F/V** Allotype
AviTag™	

Figure S2. FcgRIIIa construct sequence. Construct sequence of soluble human recombinant FcgRIIIa-158F/V with *N*-glycan sites (Asn38, Asn45, Asn74, Asn162, and Asn169) annotated in bold and signal peptide and Ig-like domains highlighted. FcgRIIIa was tagged by a 10X His-tag and AviTag™, of which the latter can be enzymatically biotinylated at the lysine (K).

Supplementary Figure 3

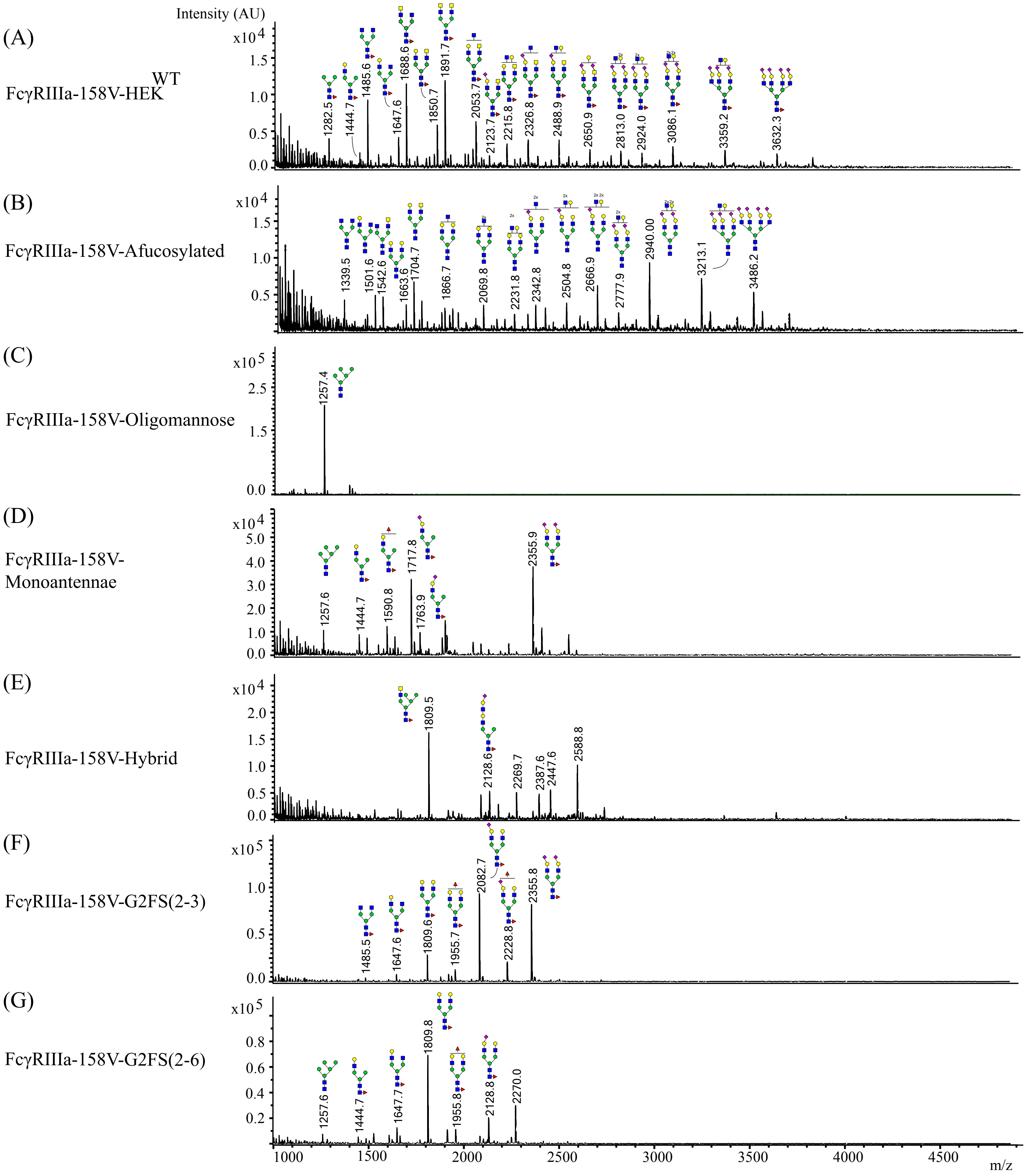

Figure S3. *N*-glycan profiling of FcgRIIIa-158F. MALDI-TOF-MS spectrum of esterified released *N*-glycans of FcgRIIIa-158V with m/z range of 1000-5000 according to Reiding *et al.* (52). **(A)** FcgRIIIa-HEK WT, **(B)** FcgRIIIa-Afucosylated by KO of *FUT8,* **(C)** FcgRIIIa-Oligomannose with (KO of *MGAT1*), **(D)** FcgRIIIa-Monoantennary (KO of *MGAT2/3*, KI *B4GALT1*), **(E)** FcgRIIIa-Hybrid *(*KO of *MAN2A1/2*), **(F)** FcgRIIIa-G2FS(2-3) (KO of *B4GALNT3/4, ΔMGAT3/4A/4B/5, ST6GAL1, FUT4*) and **(G)** FcgRIIIa-G2FS(2-6) (KO of *B4GALNT3/4, MGAT3/4A/4B/5, ST3GAL3/4/6, FUT4*). Annotation represents one possible glycan structure, which is based on gene editing signature and literature. Structures are depicted following the CFG notation (52) and the sialic acid linkage is depicted to the left or right for 2-3- or 2-6-linked sialic acid, respectively.

Supplementary Figure 4

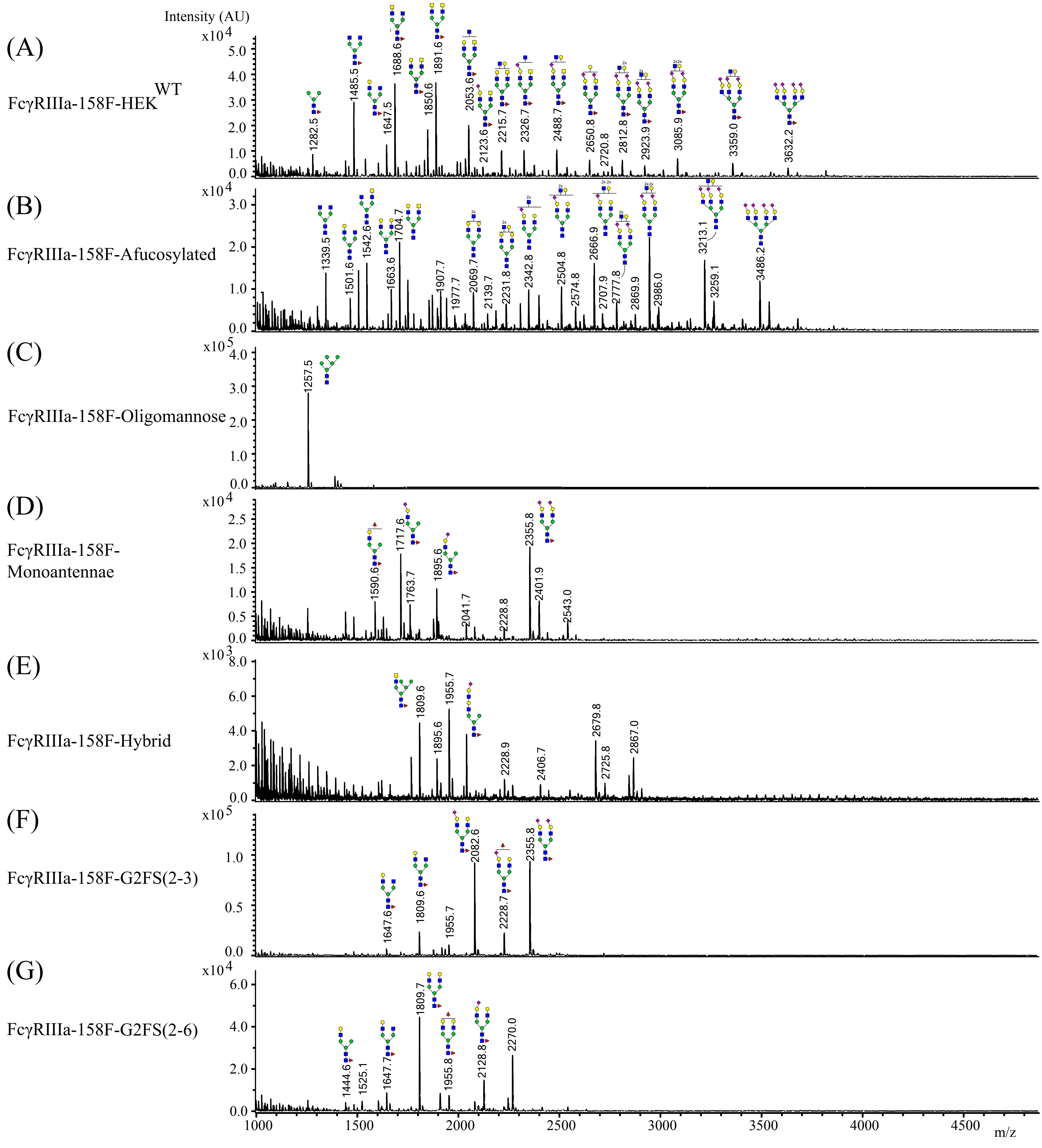

Figure S4. *N*-glycan profiling of FcgRIIIa-158V. MALDI-TOF-MS spectrum of esterified released *N*-glycans of FcgRIIIa-158V with m/z range of 1000-5000 according to Reiding *et al*. (52). **(A)** FcgRIIIa-HEK^{WT}, **(B)** FcgRIIIa-Afucosylated by KO of *FUT8*, **(C)** FcgRIIIa-Oligomannose with (KO of *MGAT1*), **(D)** FcgRIIIa-Monoantennary (KO of *MGAT2/3*, KI *B4GALT1*), **(E)** FcgRIIIa-Hybrid *(*KO of *MAN2A1/2*), **(F)** FcgRIIIa-G2FS(2-3) (KO of *B4GALNT3/4, ΔMGAT3/4A/4B/5, ST6GAL1, FUT4*) and **(G)** FcgRIIIa-G2FS(2-6) (KO of *B4GALNT3/4, MGAT3/4A/4B/5, ST3GAL3/4/6, FUT4*). Annotation represents one possible glycan structure, which is based on gene editing signature and literature. Structures are depicted following the CFG notation (51) and the sialic acid linkage is depicted to the left or right for 2-3- or 2-6-linked sialic acid, respectively.

Supplementary Figure 5

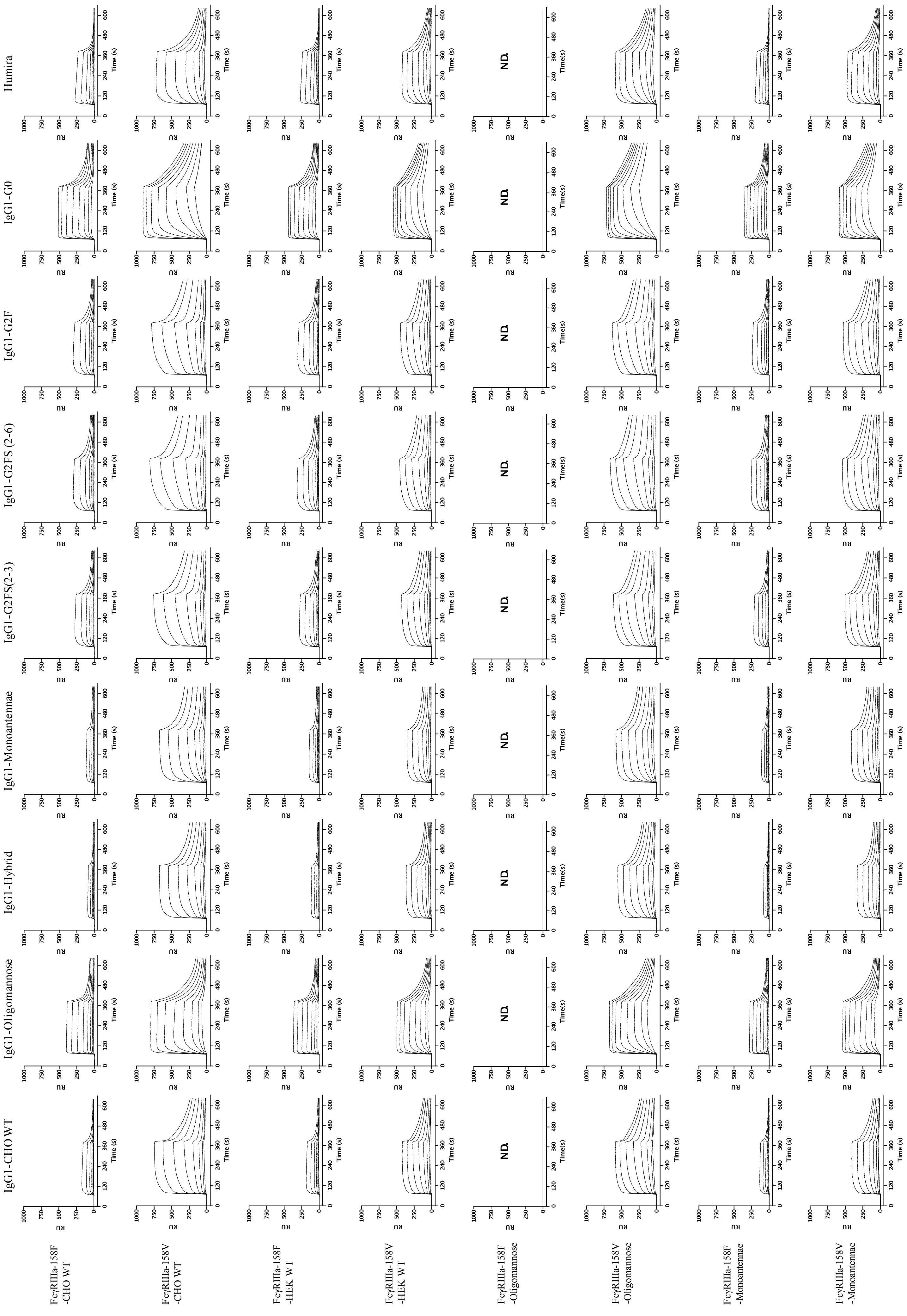

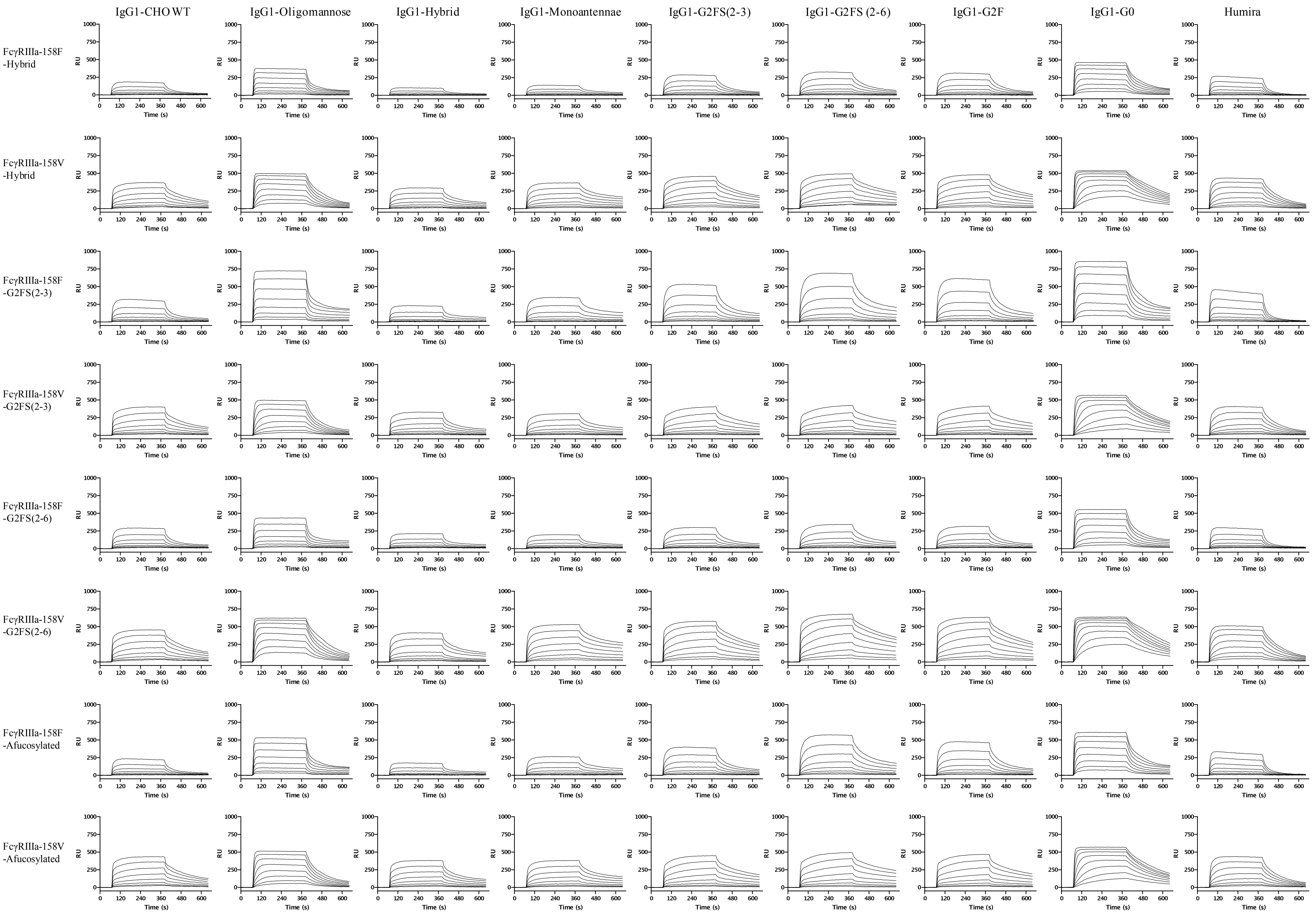

IgG1-CHO WT
IgG1-Oligomannose
IgG1-Hybrid
IgG1-Monoantennae
IgG1-G2FS(2-3)
IgG1-G2FS (2-6)
IgG1-G2F
IgG1-G0
Humira
FcγRIIIa-158F -Hybrid
FcγRIIIa-158V -Hybrid
FcγRIIIa-158F -G2FS(2-3)
FcγRIIIa-158V -G2FS(2-3)
FcγRIIIa-158F -G2FS(2-6)
FcγRIIIa-158V -G2FS(2-6)
FcγRIIIa-158F -Afucosylated
FcγRIIIa-158V -Afucosylated
RU
Time (s)

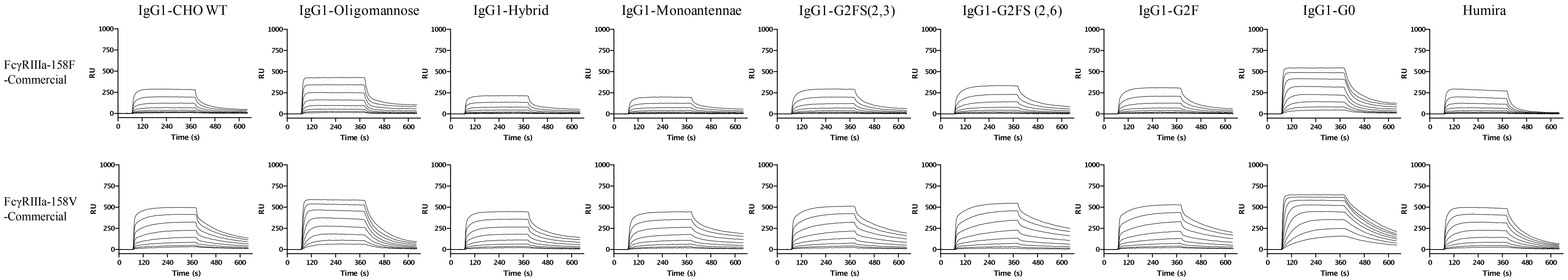

Figure S5. Sensorgrams of glycoengineered IgG1 and FcgRIIIa-158F/V binding by SPR. SPR sensorgrams are shown after biotinylated glycoengineered FcgRIIIa-158F/V was spotted in 4-fold dilutions and overflown with glycoengineered IgG1 for 8 dilution series.
